# Mycoviral Population Dynamics in Spanish Isolates of the Entomopathogenic Fungus *Beauveria bassiana*

**DOI:** 10.3390/v10120665

**Published:** 2018-11-24

**Authors:** Charalampos Filippou, Inmaculada Garrido-Jurado, Nicolai V. Meyling, Enrique Quesada-Moraga, Robert H. A. Coutts, Ioly Kotta-Loizou

**Affiliations:** 1 Department of Life Sciences, Imperial College London, London SW7 2AZ, UK; c_filippou@live.com (C.F.); g72gajui@uco.es (I.G.-J.); 2 Department of Biological and Environmental Sciences, University of Hertfordshire, Hatfield AL10 9AB, UK; r.coutts@herts.ac.uk; 3 Department of Agricultural and Forestry Sciences, University of Cordoba, 14071 Cordoba, Spain; cr2qumoe@uco.es; 4 Department of Plant and Environmental Sciences, University of Copenhagen, 1871 Frederiksberg C, Denmark; nvm@plen.ku.dk

**Keywords:** mycovirus, *Beauveria bassiana*, partitivirus, victorivirus, polymycovirus, selection pressure, recombination, transmission

## Abstract

The use of mycoviruses to manipulate the virulence of entomopathogenic fungi employed as biocontrol agents may lead to the development of novel methods to control attacks by insect pests. Such approaches are urgently required, as existing agrochemicals are being withdrawn from the market due to environmental and health concerns. The aim of this work is to investigate the presence and diversity of mycoviruses in large panels of entomopathogenic fungi, mostly from Spain and Denmark. In total, 151 isolates belonging to the genera *Beauveria*, *Metarhizium*, *Lecanicillium*, *Purpureocillium*, *Isaria*, and *Paecilomyces* were screened for the presence of dsRNA elements and 12 Spanish *B. bassiana* isolates were found to harbor mycoviruses. All identified mycoviruses belong to three previously characterised species, the officially recognised *Beauveria bassiana victorivirus 1* (BbVV-1) and the proposed Beauveria bassiana partitivirus 2 (BbPV-2) and Beauveria bassiana polymycovirus 1 (BbPmV-1); individual *B. bassiana* isolates may harbor up to three of these mycoviruses. Notably, these mycovirus species are under distinct selection pressures, while recombination of viral genomes increases population diversity. Phylogenetic analysis of the RNA-dependent RNA polymerase gene sequences revealed that the current population structure in Spain is potentially a result of both vertical and horizontal mycovirus transmission. Finally, pathogenicity experiments using the Mediterranean fruit fly *Ceratitis capitata* showed no direct correlation between the presence of any particular mycovirus and the virulence of the *B. bassiana* isolates, but illustrated potentially interesting isolates that exhibit relatively high virulence, which will be used in more detailed virulence experimentation in the future.

## 1. Introduction

Recently there has been a resurgence of interest in the use of microbial control agents as alternatives to chemical pesticides or as part of integrated pest management programs [1]. Among the fungal-based biopesticides, entomopathogenic fungi within the Ascomycota and order Hypocreales are represented in over 150 commercially available products [2], mostly based on strains from the genera *Beauveria*, *Metarhizium*, *Isaria*, and *Lecanicillium* [3]. Although entomopathogenic fungi constitute an environmentally friendly alternative to chemical pesticides, it is crucial to optimise their application in order to ensure maximum efficiency and reliability [4].

Mycoviruses are currently classified in seventeen taxa, sixteen families and one genus that does not belong to a family; eight of these taxa accommodate mycoviruses with double-stranded (ds) RNAs as their genome, including the families *Totiviridae* and *Partitiviridae*. Totiviruses have a linear non-segmented genome, while partitiviruses have two genomic segments. The members of these families encode two proteins, an RNA-dependent RNA polymerase (RdRp) required for genome replication, and a capsid protein (CP) that forms icosahedral virions (https://talk.ictvonline.org/ictv-reports/ictv_online_report). Recently, a novel virus family has been described, provisionally designated as the Polymycoviridae [5,6]. Polymycoviruses have four dsRNA segments as their genome, one encoding the RdRp, and are non-conventionally encapsidated and are as infectious as dsRNA [5]. To date, dsRNA mycoviruses are not known to have an extracellular phase in their replication cycle. They can be transmitted horizontally from one fungal strain to another via hyphal fusion (anastomosis), or vertically from parent to offspring during the formation of sexual or asexual spores. Therefore, dispersion of these mycoviruses depends entirely on their hosts’ movements. The majority of mycoviral infections have no discernible effects on the fungal host. Nevertheless, cases of hypovirulence [7,8,9], and more recently hypervirulence [5,10,11], have been reported, including an increase in growth and virulence of the entomopathogenic fungus *B. bassiana* caused by a polymycovirus [6].

The aim of the present work was to investigate the presence and diversity of mycoviruses in a selection of entomopathogenic fungal isolates belonging to the genera *Beauveria, Metarhizium, Lecanicillium, Purpureocillium*, *Isaria*, and *Paecilomyces,* derived from Spain and Denmark. Partial sequencing of the mycoviruses found and subsequent data analysis provided insights into population structure and dynamics, including selection pressures, recombination and transmission. Finally, a rapid pathogenicity screening against the Mediterranean fruit fly *Ceratitis capitata* highlighted potentially interesting isolates to be used in more detailed virulence experiments in the future.

## 2. Materials and Methods

### 2.1. Screening of Fungal Isolates and Molecular Cloning

In total, 151 isolates from two collections of entomopathogenic fungi (Table S1), one from the University of Cordoba, Spain, and one from the University of Copenhagen, Denmark, were used in the present study. The Spanish collection is kept at −80 °C following lyophilisation and the viability of the isolates is checked every two years. The Danish collection is also kept at −80 °C as conidia, suspended in 1:1 of glycerol:10% (*w*/*v*) skimmed milk. None of the isolates in this study are being used commercially as biological control agents. All isolates were grown on malt agar extract (MEA) medium at 25 °C and screened for the presence of dsRNA elements using phenol/Sevag treatment and enzymatic digestions with DNase I (Promega) and S1 nuclease (Promega) as described previously [6]. A random reverse transcriptase-chain polymerase reaction process [12] was applied for the initial identification of the dsRNAs, and target-specific primers (Table S2) were subsequently designed with the help of Primer-BLAST [13]. For fungal identification, DNA was extracted using a phenol/Sevag treatment, following disruption of the mycelia in liquid nitrogen [14], and the universal primers ITS1F (5′-CTT GGT CAT TTA GAG GAA GTA A-3′ [15] and ITS4 (5′-TCC TCC GCT TAT TGA TAT GC-3′ [16]) were used to amplify the complete sequence of internal transcribed spacer (ITS) 1, the 5.8S ribosomal RNA gene, and the internal transcribed spacer 2, flanked by the partial sequence of the 18S and 28S ribosomal RNA genes. Amplicons were then cloned using the pGEM-T Easy System (Promega, Madison, WI, USA) and transformed into X10-Gold Ultracompetent Cells (Agilent, Santa Clara, CA, USA). Plasmids were extracted using the QIAprep Spin Miniprep Kit (QIAGEN, Hilden, Germany) and at least three independent clones were sequenced for each amplicon. All sequences were deposited in the European Nucleotide Archive: BbVV-1 strain EABb 01/33-Su accession number LR028007; BbVV-1 strain EABb 01/112-Su accession number LR028008; BbVV-1 strain EABb 00/13-Su accession number LR028009; BbVV-1 strain EABb 07/06-Rf accession number LR028010; BbVV-1 strain EABb 10/57-Fil accession number LR028011; BbVV-1 strain EABb 11/01-Mg accession number LR028012; BbVV-1 strain EABb 00/11-Su accession number LR028013; BbPV-2 strain EABb 10/57-Fil accession number LR028014; BbPV-2 strain EABb 09/07-Fil accession number LR028015; BbPV-2 strain EABb 00/13-Su accession number LR028016; BbPV-2 strain EABb 11/01-Mg accession number LR028017; BbPV-2 strain EABb 07/06-Su accession number LR028018; BbPV-2 strain EABb 00/11-Su accession number LR028019; BbPmV-1 strain EABb 10/57-Fil accession number LR028020; BbPmV-1 strain EABb 10/28-Su accession number LR028021; BbPmV-1 strain EABb 00/13-Su accession number LR028022; BbPmV-1 strain EABb 00/11-Su accession number LR028023; BbPmV-1 strain EABb 07/06-Rf accession number LR028024; BbPmV-1 strain EABb 11/01-Mg accession number LR028025; BbPmV-1 strain EABb 10/01-Fil accession number LR028026; BbPmV-1 strain EABb 10/30-Fil accession number LR028027; ITS strain EABb 10/01-Fil accession number LR028032; ITS strain EABb 09/07-Fil accession number LR028033; ITS strain EABb 00/11-Su accession number LR028034; ITS strain EABb 01/112-Su accession number LR028035; ITS strain EABb 11/01-Mg accession number LR028036; ITS strain EABb 07/06-Rf accession number LR028037; ITS strain EABb 00/13-Su accession number LR028038; ITS strain EABb 10/28-Su accession number LR028039; ITS strain EABb 10/57-Fil accession number LR028040; ITS strain EABb 10/30-Fil accession number LR028041.

### 2.2. Computational and Phylogenetic Analysis

Sequence similarity searches of the GenBank, Swissprot, and EMBL databases were conducted using the BLASTx program [17]. Phylogenetic analysis of viral nucleotide sequences and p-distance calculations were performed using MEGA 6 [18], following alignment using MUSCLE as implemented by MEGA 6. Maximum likelihood phylogenetic trees were constructed using the K2 + I + G substitution model for victoriviruses, the K2 + G substitution model for partitiviruses ITS sequences and the T92 + I substitution model for polymycoviruses. The most appropriate substitution model for each set of sequences was selected using MEGA 6. The codon-based Z-test of selection on the overall average of viral sequences was also performed using MEGA 6 by computing the number of synonymous (dS) and non-synonymous (dN) substitutions per site. The probability of rejecting the null hypothesis of strict-neutrality (dN = dS) in favour of the alternative hypothesis (dN < dS or dN > dS) were calculated and *p*-values less than 0.05 were considered significant at the 5% level. The test statistic (dS–dN) was calculated and the variance of the difference was computed using the bootstrap method (100 replicates). Analyses were conducted using the Nei-Gojobori method [19]. If the number of synonymous substitutions was significantly higher than the number of non-synonymous substitutions then the population was under purifying/negative selection. Conversely, if the number of synonymous substitutions was significantly lower than the number of non-synonymous substitutions then the population was under positive selection. Recombination events were detected using RDP4 [20].

### 2.3. Insect Pathogenicity Experiments

The insect pathogenicity experiments were performed following standard protocols [21,22,23,24,25,26]. Conidial suspensions were prepared from selected isolates by scraping the conidia from Petri plates with malt extract agar (MEA) medium into a sterile aqueous solution of 0.1% Tween 80. Each conidial suspension was filtered through cheesecloth to remove the mycelial mat and adjusted to a concentration of 1.0 × 10^8^ conidia per ml. Cold-anesthetised newly emerged *C. capitata* adults were sprayed with 1 mL of the conidial suspension in a Potter Spray Tower (Burkard Scientific, Uxbridge, UK). Each repetition of 10 insects were treated separately. Control flies were treated with the same volume of a sterile aqueous solution of 0.1% Tween 80. The treated adult *C. capitata* were placed in methacrylate boxes (8 × 8 × 6 cm) containing a circular hole of 2 cm in diameter covered with a net cloth. The bioassay conditions were 26 ± 2 ℃, 50–60% RH and a photoperiod of 16:8 (L:D) h. An adult diet (0.1 g of hydrolyzed protein and 0.4 g sucrose with 1.5 mL of distilled water) and water were provided every 24 h. Three replicates of 10 insects were used and mortality was monitored daily for 8 days. Dead flies were removed daily, immediately surface-sterilised and placed in humidity chambers for observation of mycosis as outlined by Quesada-Moraga et al. (2006) [27]. Data were analyzed using a generalised linear model (distribution = binomial; link = logit) and treatment comparisons were performed applying the χ^2^ test (*p* < 0.05).

## 3. Results and Discussion

### 3.1. Presence of dsRNA Elements in B. bassiana Isolates from the Iberian Peninsula

**Figure 1:**
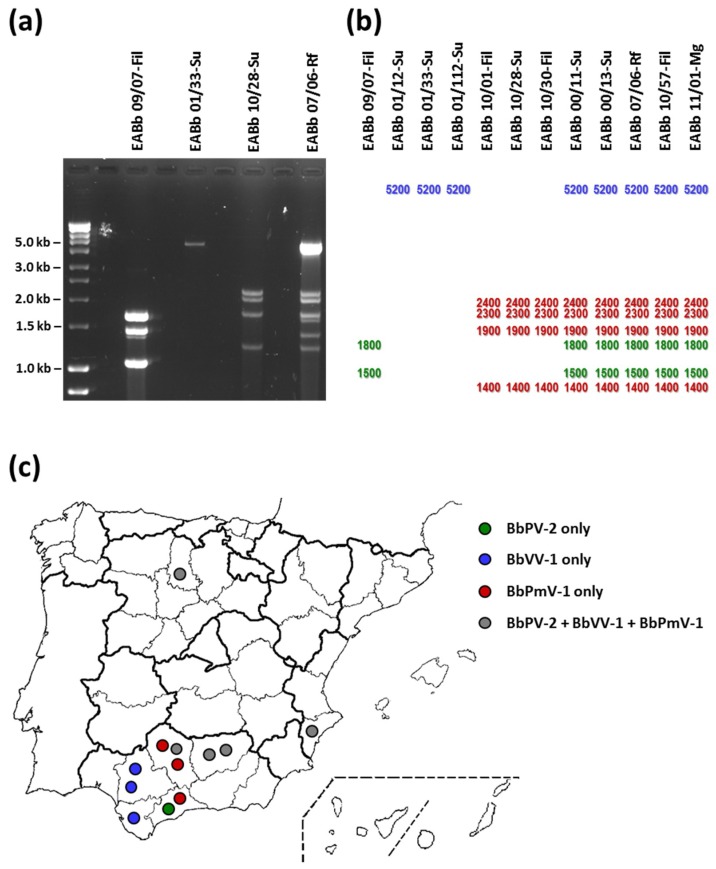
(**a**) 1% (*w*/*v*) agarose gel electrophoresis of dsRNA elements from four *Beauveria bassiana* isolates, representative of the four observed electrophoretic profiles. (**b**) Schematic representation of the observed electrophoretic profiles of dsRNA elements isolated from 12 *B. bassiana* isolates and their relative sizes, green for members of the family *Partitiviridae*, blue for members of the family *Totiviridae* and red for members of the proposed family Polymycoviridae. (**c**) Geographical distribution of mycoviruses found in Spanish *B. bassiana* isolates.

Seventy-five *Metarhizium* sp. isolates [28] and two *B. bassiana* isolates [29] from Denmark, together with seventy-four isolates mostly from the Iberian Peninsula [30], including fifty *Beauveria* sp., thirteen *Metarhizium* sp., eight *Purpureocillium lilacinum*, two *Lecanicillium attenuatum*, one *Isaria farinosa*, and one *Paecilomyces marquandii* were screened for the presence of dsRNA elements. Putative mycoviruses were discovered in twelve out of forty (30%) *B. bassiana* isolates of Spanish provenance (Table 1), collected mainly from the south of Spain (Figure 1c). Isolate EABb 01/12-Su is known to be infected by a strain of *Beauveria bassiana victorivirus 1* (BbVV-1; [6], while isolates EABb 01/33-Su and EABb 00/11-Su have previously been reported to harbour uncharacterised dsRNA elements [31]. No dsRNA elements were discovered in any of the Danish *Metarhizium* sp. or *B. bassiana* isolates, although their presence has been documented previously in populations of *Metarhizium* mostly from Brazil [32,33,34,35,36,37,38,39]. The number of isolates from other fungal species screened was very small and no dsRNA elements were found in any of these. A notable presence of dsRNA elements or mycoviruses has been reported previously for *P. lilacinum* [40], *I. farinosa* [41] and other *Isaria* sp. [41], and *Paecilomyces* sp. [37,41]. The reason behind the high prevalence of dsRNA elements in the Spanish population is not clear. In Spain, there are three products registered in the Official Register for Phytosanitary Products and Materials of the Ministry of Agriculture, Forestry and Fisheries, and commercialised based on *B. bassiana*: Naturalis-L (ATCC 74040 strain), Botanigard, and Botanigard 22 WP (GHA strain). These are used to protect fruits and vegetables, such as apple, aubergine, beans, broccoli, cherry, citrus, cauliflower, cotton, cucurbit, grapevine, kaki, lettuce, olive, pear, green pepper, potato, strawberry, and tomato, against aphids, tephritids, thrips, and whiteflies. It should be noted that isolates ATCC 74040 and GHA are both virus-free [6], therefore the high prevalence of dsRNA elements cannot be explained by their potential introduction in the ecosystem via these biological control agents.

### 3.2. Mixed Infections of B. bassiana Isolates with Up to Three Different Mycoviruses

Following agarose electrophoresis of the purified dsRNA elements from all twelve *B. bassiana* isolates, four distinct electrophoretic profiles were noted as depicted in Figure 1a,b. One isolate harbours two dsRNAs 1–2 kbp in size, potentially a member of the family *Partitiviridae*, together with a smaller dsRNA which is probably a satellite RNA. Three isolates harbor a sole large dsRNA approximately 5 kbp in size, potentially a member of the family *Totiviridae*. Three isolates exhibit a banding pattern reminiscent of the proposed family Polymycoviridae. The rest—including isolate EABb 11/01-Mg from the pine sawyer beetle *Monochamus galloprovincialis* that acts as a vector for the parasitic nematode *Bursaphelenchus xylophilus*, causative agent of pine wilt–contain a combination of the above, suggesting the presence of multiple mycovirus infections. Since the profiles were identical to those observed previously [6], and initial molecular characterisation experiments revealed the presence of mycoviruses very similar to already fully sequenced strains, three oligonucleotide primer pairs were used to amplify part of the RdRp genes of BbVV-1 [31], Beauveria bassiana partitivirus 2 (BbPV-2) [6] and Beauveria bassiana polymycovirus 1 (BbPmV-1) [6]. All amplicons were cloned and sequenced and the presence of quasispecies, highly similar but not necessarily identical (Figure S1) strains, of the aforementioned mycoviruses in the fungal isolates was confirmed. Based on this and previous studies, all three viruses appear to be widespread in Spain. BbVV-1-like and BbPmV-1-like strains were discovered in eight out of forty (20%) Spanish *B. bassiana* isolates, while six out of forty (15%) harbor BbPV-2-like strains. To date, BbVV-1-like and BbPmV-1-like strains have been discovered exclusively in Spain, while BbPV-2-like strains have also been found in Asia and South America [6,31]. According to Andino and Domingo (2015), “viral quasispecies are defined as collections of closely related viral genomes subjected to a continuous process of genetic variation, competition among the variants generated, and selection of the most fit distributions in a given environment” [42]. Quasispecies are the result of the high mutation rates of the error-prone RdRps, which characterise all RNA viruses and lead to populations of mutants instead of identical viral genomes [43]. The quasispecies theory has been employed to better understand RNA viral pathogens, e.g., human immunodeficiency virus [44,45], their interactions within the population and with their host, and to design appropriate therapeutic approaches [46]. To our knowledge, this is the first time viral quasispecies have been discussed in the context of mycoviruses. However, since the potential effects of the mycoviruses on their hosts and the molecular mechanisms that underpin those are understudied, it is more difficult to appreciate the significance of this observation, for instance regarding the use of mycoviruses as enhancers of mycoinsecticides. 

### 3.3. Selection Pressures and Recombination Events within the Viral Genome

Interestingly, while BbVV-1-like and BbPV-2-like strains are under positive selection, as indicated by a codon-based Z-test (*p* < 0.05; for BbVV-1-like strains: dN–dS = 6.28; 12 nucleotide sequences analysed, for BbPV-2-like strains: dN–dS = 3.17; 7 nucleotide sequences analysed), BbPmV-1-like strains appear to be under purifying or negative selection (*p* < 0.05; dS–dN = 17.30; 9 nucleotide sequences analysed). Positive selection is inferred by the significantly higher abundance of non-synonymous substitutions as compared to synonymous substitutions in the RdRp gene sequences and indicate an evolving viral population where novel phenotypic traits are evaluated favourably [47]. In contrast, purifying or negative selection does not allow for non-synonymous nucleotide substitutions that alter the protein sequence, which are then eliminated from the population [47], because they are presumably deleterious to the mycovirus. The reason why the polymycovirus RdRps are less tolerant to new variants as compared to those of victoriviruses and partitiviruses is debatable and may not be limited to the functionality of the protein as the enzyme responsible for viral replication. For instance, it has been speculated that the BbPmV-1 RdRp may physically interact with at least some of the other viral proteins (the putative scaffold protein, the methyl transferase, and/or the protein coating the viral genome [6]) and mutations that abolish these interactions would not be tolerated. Moreover, BbPmV-1 causes an increase in growth and virulence of *B. bassiana* [6], the molecular mechanisms of which are as yet unknown. However, hypervirulence may be mediated at least partially via virus-host protein interactions whose disruption is not favoured, since it deprives the fungal host and consequently the virus from this advantage. Finally, another mechanism contributing to the genetic variation of viral quasispecies is recombination [43] and one recombination event was detected in the case of BbPmV-1-like viruses: BbPmV-1 strain EABb 10/30-Fil is a recombinant of BbPmV-1 strain EABb 11/01-Mg (major parent) and BbPmV-1 strain EABb 10/28-Su (minor parent). More specifically, nucleotides 1-625 and 854-876 of the BbPmV-1 strain EABb 10/30-Fil amplified segment are derived from the major parent and nucleotides 626-853 are derived from the minor parent (Figure S2). Notably, this observation suggests that the two BbPmV-1-like viruses must have been simultaneously present in the same fungal isolate for the recombination event to take place. Subsequently, the competition among the three BbPmV-1-like viruses would have led to the loss of the two parents and the establishment of the recombinant. It is feasible that recombination events that transfer large fragments of the protein potentially optimised for their function(s) are more tolerable for polymycoviruses than single amino acid substitutions and therefore an appropriate mechanism for increasing diversity.

### 3.4. Evidence of Vertical and Horizontal Transmission of Mycoviruses

**Figure 2:**
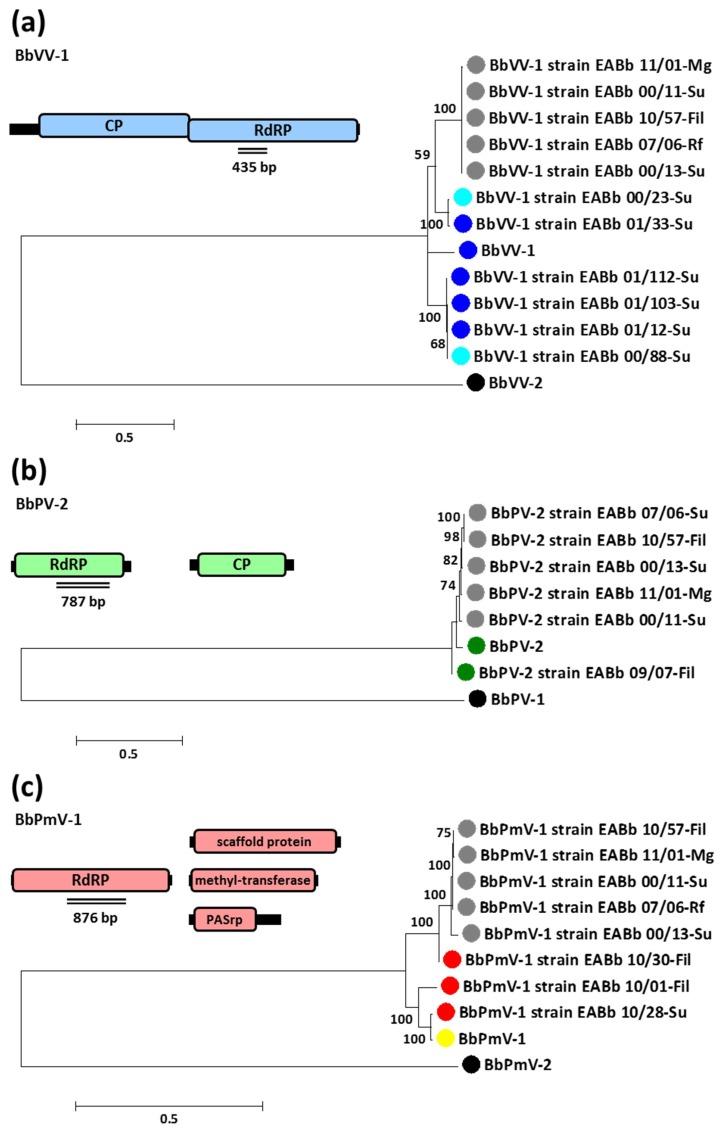
Maximum likelihood phylogenetic trees created based on the alignment of RdRp sequences of (**a**) members of the family *Partitiviridae*, (**b**) members of the family *Totiviridae* and (**c**) members of the proposed family Polymycoviridae infecting *Beauveria bassiana*. At the end of the branches: grey circles indicate that the *B. bassiana* isolate is infected with all three BbPV-2-like, BbVV-1-like, and BbPmV-1-like viruses; single infections of *B. bassiana* isolate is indicated by blue circles for BbVV-1-like virus; green circles for BbPV-2-like virus; red circles for BbPmV-1-like virus; turquoise circles for BbVV-1-like virus and a partitivirus [6]; yellow circles indicate that the *B. bassiana* isolate is infected with a BbPmV-1-like virus and a unirnavirus [57]; black circles indicate the outgroup.

Since the viral sequences obtained were all very similar but not identical to each other, phylogenetic analysis was conducted to determine their evolutionary relationships and three phylogenetic trees were constructed for BbVV-1-like, BbPV-2-like, and BbPmV-1-like strains, respectively (Figure 2). In all three cases, it is evident that the mycoviruses in the fungal isolates with triple infections are more closely related to each other than to any of the other quasispecies investigated and, at least in the case of BbVV-1-like and BbPmV-1-like strains, they form distinct clusters. This was confirmed with the p-distance matrices (Figure S1), revealing that the co-infecting viral strains are often identical. Since their genomes are highly similar, the implication is that these three mycoviruses are transmitted simultaneously as a complex, either vertically or horizontally, from parent to offspring or from one fungal strain to another. In order to clarify the mode of transmission, the evolutionary relationships among the 12 *B. bassiana* isolates was examined and a phylogenetic tree was constructed using the ITS sequences (Figure S3). There are very few nucleotide substitutions noted among the ITS sequences, therefore the *B. bassiana* isolates are evolutionarily very close to each other. Nevertheless, the isolates harbouring the triple infections no longer cluster together, suggesting the possibility of one horizontal transmission event for the three viruses between more distantly related isolates, while vertical transmission is the most likely explanation between closely related isolates. Vegetative incompatibility studies have been performed in the past on *Beauveria* populations [48,49], revealing a large number of vegetative compatibility groups (VCGs) and a low frequency of genetic exchange. However, specifically regarding mycovirus transmission, different levels of (in)compatibility between fungal strains have been reported [50], and therefore it is feasible that, even if the Spanish *B. bassiana* population is similar to the ones described previously [48,49], this would not prevent spread of the mycoviruses, which is not necessarily constricted by genetic compatibility between fungal strains. As an ascomycete, *B. bassiana* has the capacity to produce both asexual spores, conidia, and sexual spores, and ascospores. However, the sexual stage is rarely observed and *B. bassiana*, being heterothallic, does not possess within a single individual the resources to reproduce sexually, but requires two distinct individuals of opposite mating types [51]. Our difficulties with eliminating *B. bassiana* mycoviruses in the past [6] suggest that they are easily transmitted during conidiation, while studies on other ascomycetes show that transmission via ascospores is variable and often less efficient [52,53,54,55,56]. 

### 3.5. Pathogenicity of Virus-Infected B. Bassiana against the Mediterranean Fruit Fly. 

A preliminary bulk assessment of the variability in virulence among mycovirus infected *B. bassiana* isolates was performed using adult *C. capitata* as a model system. Significant differences in total mortality were found between the isolates [χ^2^ (13) = 130.7, *p* < 0.001]. The total mortality values ranged between 80.0% and 100.0% for EABb 10/30-Fil and for the isolates EABb 01/112-Su, EABb 07/06-Rf, EABb 09/07-Fil, and EABb 11/01-Mg, respectively (Table 2). Regarding cadavers producing fungal outgrowth, significant differences were also found between the isolates [χ^2^ (13) = 92.4, *p* < 0.001]. This mortality ranged between 50.0% and 90.0% for EABb 01/112-Su and EABb 07/06-Rf, respectively. No mortality with fungal outgrowth was observed in the control. The average survival times ranged from 7.5 days for the controls and 4.2 days for EABb 01/112-Su (Table 2). Interestingly, there appeared to be no correlation between the presence of specific mycoviruses and/or mycovirus complexes and the virulence of *B. bassiana* isolates. Additionally, some of the isolates with triple infections caused mortalities higher than 95.0%, suggesting that their heavy viral burdens did not impair pathogenicity. This may be explained by potential hypervirulence caused by the mycoviruses, especially strains of BbPmV-1, as shown previously [6]. Despite this previous demonstration of hypervirulence, it remains unclear if mycovirus infections in *B. bassiana* populations provide any adaptive advantage over wild-types. The absence of mycoviruses in the sampled European populations of other *Beauveria* and *Metarhizium* sp. Indicates, that for these species of entomopathogenic fungi, mycoviruses are not or at least rarely prevalent. Future experiments focusing on curing the *B. bassiana* isolates from the mycovirus infection and comparing the isogenic lines in terms of e.g., growth and germination, UV-tolerance, spore production, and pathogenicity against a range of insects will shed light on any selective advantage or related cost of mycovirus infections in entomopathogenic fungi. 

## Figures and Tables

**Table 1:** Mycovirus-infected *B. bassiana* isolates, their habitat and Spanish location.

Species	Isolate	Habitat	Location	Mycovirus *
*B. bassiana*	EABb 00/11-Su	Soil (scrubland)	Jaen	BbVV-1 + BbPV-2 + BbPmV-1
*B. bassiana*	EABb 00/13-Su	Soil (woodland)	Jaen	BbVV-1 + BbPV-2 + BbPmV-1
*B. bassiana*	EABb 01/12-Su	Soil (scrubland)	Seville	BbVV-1
*B. bassiana*	EABb 01/33-Su	Soil (olive grove)	Cadiz	BbVV-1
*B. bassiana*	EABb 01/112-Su	Soil (wheat field)	Seville	BbVV-1
*B. bassiana*	EABb 07/06-Rf	*Rhynchophorus ferrugineus*	Alicante	BbVV-1 + BbPV-2 + BbPmV-1
*B. bassiana*	EABb 09/07-Fil	Phylloplane (meadow)	Malaga	BbPV-2
*B. bassiana*	EABb 10/01-Fil	Phylloplane (olive grove)	Malaga	BbPmV-1
*B. bassiana*	EABb 10/28-Su	Soil (olive grove)	Cordoba	BbPmV-1
*B. bassiana*	EABb 10/30-Fil	Phylloplane (olive grove)	Cordoba	BbPmV-1
*B. bassiana*	EABb 10/57-Fil	Phylloplane (meadow)	Cordoba	BbVV-1 + BbPV-2 + BbPmV-1
*B. bassiana*	EABb 11/01-Mg	*Monochamus galloprovincialis*	Palencia	BbVV-1 + BbPV-2 + BbPmV-1

* BbVV-1: Beauveria bassiana victorivirus 1; BbPV-2: Beauveria bassiana partitivirus 2; BbPmV-1: Beauveria bassiana polymycovirus 1.

**Table 2:** Insecticidal activity of mycovirus infected *B*. *bassiana* isolates to new emerged *C. capitata* adults inoculated with a suspension of 1.0 × 10^8^ conidia mL^−1^.

Treatment *	Mortality (Mean ± SE)% **		Kaplan-Meier Survival Analysis
	Total Mortality	Fungal Outgrowth	AST *** (d, Mean ± SE)
**Control**	13.3 ± 3.3 a	0.0 ± 0.0 a	7.5 ± 0.3 a
EABb 10/30-Fil 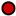	80.0 ± 0.0 b	53.3 ± 3.3 b	5.5 ± 0.4 b
EABb 01/33-Su 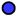	86.7 ± 3.3 b	76.7 ± 8.8 c	5.4 ± 0.3 b
EABb 00/11-Su 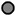	90.0 ± 0.0 b	70.0 ± 10.0 b	5.2 ± 0.4 bc
EABb 00/13-Su 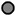	90.0 ± 5.8 b	66.6 ± 12.0 b	5.1 ± 0.3 bc
EABb 01/12-Su 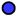	93.3 ± 6.7 bc	76.7 ± 8.8 c	4.5 ± 0.4 c
EABb 10/01-Fil 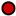	93.3 ± 3.3 bc	80.0 ± 10.0 c	5.3 ± 0.3 bc
EABb 10/28-Su 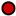	96.7 ± 3.3 c	63.3 ± 18.6 b	4.7 ± 0.4 bc
EABb 10/57-Fil 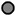	96.7 ± 3.3 c	70.0 ± 15.2 b	5.0 ± 0.4 bc
EABb 01/112-Su 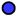	100.0 ± 0.0 c	50.0 ± 10.0 b	4.2 ± 0.4 c
EABb 07/06-Rf 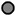	100.0±0.0 c	90.0 ± 0.0 c	4.8 ± 0.3 c
EABb 09/07-Fil 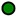	100.0±0.0 c	83.3 ± 6.7 c	5.2 ± 0.3 bc
EABb 11/01-Mg 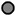	100.0±0.0 c	70.0 ± 5.8 b	5.1 ± 0.1 bc

* Grey circles indicate that the isolate is infected with all three BbPV-2-like, BbVV-1-like, and BbPmV-1-like viruses; single infections of isolates are indicated by blue circles for BbVV-1-like virus, green circles for BbPV-2-like virus and red circles for BbPmV-1-like virus. ** Means within columns with the same letter are not significantly different (χ^2^ test, *p* ≤ 0.05) according to the generalised linear model. *** AST: Average survival time was limited to 8 days. Means within columns with the same letter are not significantly different (*p* ≤ 0.05) according to the Log-Rank test.

## Supplementary Materials

The following are available online at http://www.mdpi.com/1999-4915/10/12/665/s1. Figure S1. Pairwise distance matrix created based on the RdRp sequences of chrysoviruses and related viruses; (A) members of the family *Partitiviridae*, (B) members of the family *Totiviridae* and (C) members of the proposed family Polymycoviridae infecting *Beauveria bassiana*. Figure S2: Schematic representation of potential recombination events between two BbPmV-1 strains: BbPmV-1 strain EABb 10/30-Fil is a recombinant of BbPmV-1 strain EABb 11/01-Mg (major parent) and BbPmV-1 strain EABb 10/28-Su (minor parent). Recombination breakage sites are indicated. Figure S3. Maximum likelihood phylogenetic tree created based on the alignment of ITS sequences of the 12 mycovirus infected *Beauveria bassiana* isolates. At the end of the branches: grey circles indicate that the *B. bassiana* isolate is infected with all three BbPV-2-like, BbVV-1-like, and BbPmV-1-like viruses; blue circles indicate that the *B. bassiana* isolate is exclusively infected with a BbVV-1-like virus; green circles indicate that the *B. bassiana* isolate is exclusively infected with a BbPV-2-like virus; red circles indicate that the *B. bassiana* isolate is exclusively infected with a BbPmV-1-like virus. Table S1. Fungal isolates screened for the presence of mycoviruses. Table S2. Oligonucleotide primers used for viral sequence amplification.

## References

[1] Chandler D., Bailey A.S., Tatchell G.M., Davidson G., Greaves J., Grant W.P. (2011). The development, regulation and use of biopesticides for integrated pest management. Philos. Trans. R. Soc. Lond. B Biol. Sci..

[2] De Faria M.R., Wraight S.P. (2007). Mycoinsecticides and Mycoacaricides: A comprehensive list with worldwide coverage and international classification of formulation types. Biol. Control.

[3] Lacey L.A., Grzywacz D., Shapiro-Ilan D.I., Frutos R., Brownbridge M., Goettel M.S. (2015). Insect pathogens as biological control agents: Back to the future. J. Invertebr. Pathol..

[4] St. Leger R.J., Wang C. (2010). Genetic engineering of fungal biocontrol agents to achieve greater efficacy against insect pests. Appl. Microbiol. Biotechnol..

[5] Kanhayuwa L., Kotta-Loizou I., Özkan S., Gunning A.P., Coutts R.H.A. (2015). A novel mycovirus from *Aspergillus fumigatus* contains four unique dsRNAs as its genome and is infectious as dsRNA. Proc. Natl. Acad. Sci. USA.

[6] Kotta-Loizou I., Coutts R.H.A. (2017). Studies on the virome of the entomopathogenic fungus *Beauveria bassiana* reveal novel dsRNA elements and mild hypervirulence. PLoS Pathog..

[7] Nuss D.L. (1992). Biological control of chestnut blight: An example of virus-mediated attenuation of fungal pathogenesis. Microbiol. Rev..

[8] Bhatti M.F., Jamal A., Petrou M.A., Cairns T.C., Bignell E.M., Coutts R.H.A. (2011). The effects of dsRNA mycoviruses on growth and murine virulence of *Aspergillus fumigatus*. Fungal Genet. Biol..

[9] Liu L., Xie J., Cheng J., Fu Y., Li G., Yi X., Jiang D. (2014). Fungal negative-stranded RNA virus that is related to bornaviruses and nyaviruses. Proc. Natl. Acad. Sci. USA.

[10] Özkan S., Coutts R.H.A. (2015). *Aspergillus fumigatus* mycovirus causes mild hypervirulent effect on pathogenicity when tested on *Galleria mellonella*. Fungal Genet. Biol..

[11] Okada R., Ichinose S., Takeshita K., Urayama S.I., Fukuhara T., Komatsu K., Arie T., Ishihara A., Egusa M., Kodama M. (2018). Molecular characterization of a novel mycovirus in *Alternaria alternata* manifesting two-sided effects: Down-regulation of host growth and up-regulation of host plant pathogenicity. Virology.

[12] Froussard P. (1992). A random-PCR method (rPCR) to construct whole cDNA library from low amounts of RNA. Nucleic Acids Res..

[13] Ye J., Coulouris G., Zaretskaya I., Cutcutache I., Rozen S., Madden T. (2012). Primer-BLAST: A tool to design target-specific primers for polymerase chain reaction. BMC Bioinform..

[14] Raeder U., Broda P. (1985). Rapid preparation of DNA from filamentous fungi. Lett. Appl. Microbiol..

[15] Gardes M., Bruns T.D. (1993). ITS primers with enhanced specificity for basidiomycetes—Application to the identification of mycorrhizae and rusts. Mol. Ecol..

[16] White T.J., Bruns T.D., Lee S.B., Taylor J.W., Innis M.A., Gelfand D.H., Sninsky J.J., White T.J. (1990). Amplification and direct sequencing of fungal ribosomal RNA genes for phylogenetics. PCR Protocols: A Guide to Methods and Applications.

[17] Altschul S.F., Madden T.L., Schäffer A.A., Zhang J., Zhang Z., Miller W., Lipman D.J. (1997). Gapped BLAST and PSI-BLAST: A new generation of protein database search programs. Nucleic Acids Res..

[18] Tamura K., Stecher G., Peterson D., Filipski A., Kumar S. (2013). MEGA6: Molecular Evolutionary Genetics Analysis version 6.0. Mol. Biol. Evol..

[19] Nei M., Gojobori T. (1986). Simple methods for estimating the numbers of synonymous and nonsynonymous nucleotide substitutions. Mol. Biol. Evol..

[20] Martin D.P., Murrell B., Golden M., Khoosal A., Muhire B. (2015). RDP4: Detection and analysis of recombination patterns in virus genomes. Virus Evol..

[21] Garrido-Jurado I., Torrent J., Barrón V., Corpas A., Quesada-Moraga E. (2011). Soil properties affect the availability, movement, and virulence of entomopathogenic fungi conidia against puparia of *Ceratitis capitata* (Diptera: Tephritidae). Biol. Control.

[22] Garrido-Jurado I., Valverde-García P., Quesada-Moraga E. (2011). Use of a multiple logistic regression model to determine the effects of soil moisture and temperature on the virulence of entomopathogenic fungi against pre-imaginal Mediterranean fruit fly *Ceratitis capitata*. Biol. Control.

[23] Quesada-Moraga E., Valverde-García P., Garrido-Jurado I. (2012). The effect of temperature and soil moisture on the development of the preimaginal Mediterranean fruit fly (Diptera: Tephritidae). Environ. Entomol..

[24] Yousef M., Garrido-Jurado I., Quesada-Moraga E. (2014). One *Metarhizium brunneum* strain, two uses to control *Ceratitis capitata* (Diptera: Tephritidae). J. Econ. Entomol..

[25] Yousef M., Aranda-Valera E., Quesada-Moraga E. (2018). Lure-and-infect and lure-and-kill devices based on *Metarhizium brunneum* for spotted wing *Drosophila* control. J. Pest Sci..

[26] Yousef M., Alba-Ramírez C., Garrido Jurado I., Mateu J., Raya Díaz S., Valverde-García P., Quesada-Moraga E. (2018). Metarhizium brunneum (Ascomycota; Hypocreales) Treatments Targeting Olive Fly in the Soil for Sustainable Crop Production. Front. Plant Sci..

[27] Quesada-Moraga E., Ruiz-García A., Santiago-Alvarez C. (2006). Laboratory evaluation of entomopathogenic fungi *Beauveria bassiana* and *Metarhizium anisopliae* against puparia and adults of *Ceratitis capitata* (Diptera: Tephritidae). J. Econ. Entomol..

[28] Steinwender B.M., Enkerli J., Widmer F., Eilenberg J., Thorup-Kristensen K., Meyling N.V. (2014). Molecular diversity of the entomopathogenic fungal Metarhizium community within an agroecosystem. J. Invertebr. Pathol..

[29] Meyling N.V., Lübeck M., Buckley E.P., Eilenberg J., Rehner S.A. (2009). Community composition, host-range and genetic structure of the fungal entomopathogen *Beauveria* in adjoining agricultural and semi-natural habitats. Mol. Ecol..

[30] Garrido-Jurado I., Fernández-Bravo M., Campos C., Quesada-Moraga E. (2015). Diversity of entomopathogenic Hypocreales in soil and phylloplanes of five Mediterranean cropping systems. J. Invertebr. Pathol..

[31] Herrero N., Dueñas E., Quesada-Moraga E., Zabalgogeazcoa I. (2012). Prevalence and diversity of viruses in the entomopathogenic fungus *Beauveria bassiana*. Appl. Environ. Microb..

[32] Leal S.C.M., Bertioli D.J., Ball B.V., Butt T.M. (1994). Presence of double-stranded RNAs and virus-like particles in the entomopathogenic fungus *Metarhizium anisopliae*. Biocontrol Sci. Technol..

[33] Bogo M.R., Queiroz M.V., Silva D.M., Giménez M.P., Azevedo J.L., Schrank A. (1996). Double-stranded RNA and isometric virus-like particles in the entomopathogenic fungus *Metarhizium anisopliae*. Mycol. Res..

[34] Melzer M.J., Bidochka M.J. (1998). Diversity of double-stranded RNA viruses within populations of entomopathogenic fungi and potential implications for fungal growth and virulence. Mycologia.

[35] Martins M.K., Furlaneto M.C., Sosa-Gomes D.R., Faria M.R., Fungaro M.H.P. (1999). Double-stranded RNA in the entomopathogenic fungus *Metarhizium flavoviride*. Curr. Genet..

[36] De la Paz Giménez-Pecci M., Bogo M., Santi L., De Moraes C.K., Corrêa C.T., Vainstein M.H., Schrank A. (2002). Characterization of mycoviruses and analyses of chitinase secretion in the biocontrol fungus *Metarhizium anisopliae*. Curr. Microbiol..

[37] Tiago P.V., Fungaro M.H., de Faria M.R., Furlaneto M.C. (2004). Effects of double-stranded RNA in *Metarhizium anisopliae* var. *acridum* and *Paecilomyces fumosoroseus* on protease activities, conidia production, and virulence. Can. J. Microbiol..

[38] Perinotto W.M.S., Golo P.S., Coutinho Rodrigues C.J.B., Sá F.A., Santi L., Beys da Silva W.O., Junges A., Vainstein M.H., Schrank A., Salles C.M.C. (2014). Enzymatic activities and effects of mycovirus infection on the virulence of *Metarhizium anisopliae* in *Rhipicephalus microplus*. Vet. Parasitol..

[39] Santos V., Mascarin G.M., da Silva Lopes M., Alves M.C.D.F., Rezende J.M., Gatti M.S.V., Dunlap C.A., Júnior Í.D. (2017). Identification of double-stranded RNA viruses in Brazilian strains of *Metarhizium anisopliae* and their effects on fungal biology and virulence. Plant Gene.

[40] Herrero N. (2016). A novel monopartite dsRNA virus isolated from the entomopathogenic and nematophagous fungus *Purpureocillium lilacinum*. Arch. Virol..

[41] Inglis P.W., Valadares-Inglis M.C. (1997). Rapid isolation of double-stranded RNAs from entomopathogenic species of the fungus *Paecilomyces* using a commercial minicolumn system. J. Virol. Methods.

[42] Andino R., Domingo E. (2015). Viral quasispecies. Virology.

[43] Domingo E., Perales C. (2018). Quasispecies and virus. Eur. Biophys. J..

[44] Briones C., Domingo E. (2008). Minority report: Hidden memory genomes in HIV-1 quasispecies and possible clinical implications. AIDS Rev..

[45] Rios A. (2018). Fundamental challenges to the development of a preventive HIV vaccine. Curr. Opin. Virol..

[46] Lauring A.S., Andino R. (2010). Quasispecies theory and the behavior of RNA viruses. PLoS Pathog..

[47] Domingo E., Sheldon J., Perales C. (2012). Viral quasispecies evolution. Microbiol. Mol. Biol. Rev..

[48] Couteaudier Y., Viaud M. (1997). New insights into population structure of *Beauveria bassiana* with regard to vegetative compatibility groups and telomeric restriction fragment length polymorphisms. FEMS Microbiol. Ecol..

[49] Castrillo L.A., Griggs M.H., Vandenberg J.D. (2004). Vegetative compatibility groups in indigenous and mass-released strains of the entomopathogenic fungus *Beauveria bassiana*: Likelihood of recombination in the field. J. Invertebr. Pathol..

[50] Deng F., Melzer M.S., Boland G.J. (2002). Vegetative compatibility and transmission of hypovirulence-associated dsRNA in *Sclerotinia homoeocarpa*. Can. J. Plant Pathol..

[51] Xiao G., Ying S.H., Zheng P., Wang Z.L., Zhang S., Xie X.Q., Shang Y., St Leger R.J., Zhao G.P., Wang C. (2012). Genomic perspectives on the evolution of fungal entomopathogenicity in *Beauveria bassiana*. Sci. Rep..

[52] Coenen A., Kevei F., Hoekstra R.F. (1997). Factors affecting the spread of double-stranded RNA viruses in *Aspergillus nidulans*. Genet. Res..

[53] Anagnostakis S.L., Chen B., Geletka L.M., Nuss D.L. (1998). Hypovirus transmission to ascospore progeny by field-released transgenic hypovirulent strains of *Cryphonectria parasitica*. Phytopathology.

[54] Varga J., Rinyu E., Kevei E., Tóth B., Kozakiewicz Z. (1998). Double-stranded RNA mycoviruses in species of *Aspergillus* sections Circumdati and Fumigati. Can. J. Microbiol..

[55] Deng F., Allen T.D., Hillman B.I., Nuss D.L. (2007). Comparative analysis of alterations in host phenotype and transcript accumulation following hypovirus and mycoreovirus infections of the chestnut blight fungus *Cryphonectria parasitica*. Eukaryot. Cell.

[56] Chun S.J., Lee Y.H. (2009). Inheritance of dsRNAs in the rice blast fungus, *Magnaporthe grisea*. FEMS Microbiol. Lett..

[57] Kotta-Loizou I., Sipkova J., Coutts R.H.A. (2015). Identification and sequence determination of a novel double-stranded RNA mycovirus from the entomopathogenic fungus *Beauveria bassiana*. Arch. Virol..

