# Supplementary material for: Mycoviral Population Dynamics in Spanish Isolates of the Entomopathogenic Fungus Beauveria bassiana
Source: Viruses. 2018 Nov 24;10(12):665. doi: 10.3390/v10120665 (PMC6315922; doi:10.3390/v10120665)
Supplement: Supplementary file 1 [file viruses-10-00665-s001.zip › SI/Figure_S1.docx]

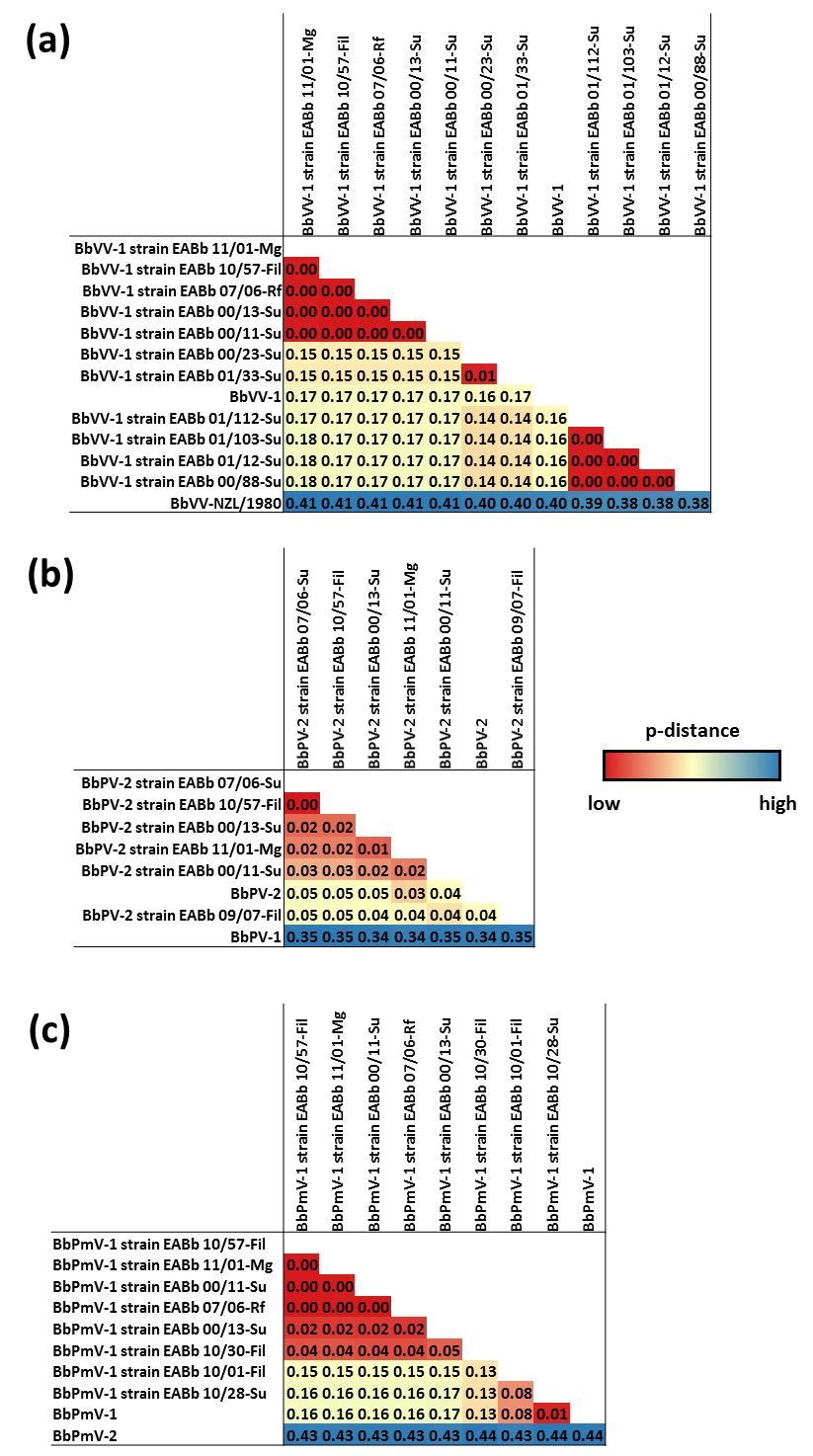


**Figure S2.** Pairwise distance matrix created based on the RdRp sequences of chrysoviruses and related viruses; **(a)** members of the family *Partitiviridae*, **(b)** members of the family *Totiviridae* and **(c)** members of the proposed family Polymycoviridae infecting *Beauveria bassiana*.
