# Supplementary material for: Mycoviral Population Dynamics in Spanish Isolates of the Entomopathogenic Fungus Beauveria bassiana
Source: Viruses. 2018 Nov 24;10(12):665. doi: 10.3390/v10120665 (PMC6315922; doi:10.3390/v10120665)
Supplement: Supplementary file 1 [file viruses-10-00665-s001.zip › SI/Figure_S2.docx]

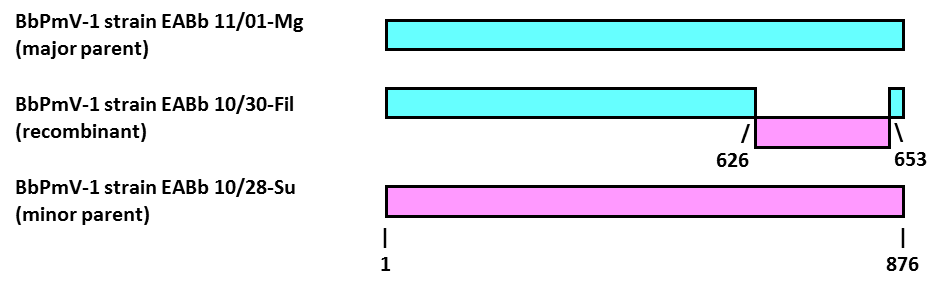


**Figure S1:** Schematic representation of potential recombination events between two BbPmV-1 strains: BbPmV-1 strain EABb 10/30-Fil is a recombinant of BbPmV-1 strain EABb 11/01-Mg (major parent) and BbPmV-1 strain EABb 10/28-Su (minor parent). Recombination breakage sites are indicated.
