# Supplementary material for: Mycoviral Population Dynamics in Spanish Isolates of the Entomopathogenic Fungus Beauveria bassiana
Source: Viruses. 2018 Nov 24;10(12):665. doi: 10.3390/v10120665 (PMC6315922; doi:10.3390/v10120665)
Supplement: Supplementary file 1 [file viruses-10-00665-s001.zip › SI/Figure_S3.docx]

**Figure S3.** Maximum likelihood phylogenetic tree created based on the alignment of ITS sequences of the twelve mycovirus infected *B. bassiana* isolates. At the end of the branches: grey circles indicate that the *B. bassiana* isolate is infected with all three BbPV-2-like, BbVV-1-like and BbPmV-1-like viruses; blue circles indicate that the *B. bassiana* isolate is exclusively infected with a BbVV-1-like virus; green circles indicate that the *B. bassiana* isolate is exclusively infected with a BbPV-2-like virus; red circles indicate that the *B. bassiana* isolate is exclusively infected with a BbPmV-1-like virus.
