# Supplementary material for: Mycoviral Population Dynamics in Spanish Isolates of the Entomopathogenic Fungus Beauveria bassiana
Source: Viruses. 2018 Nov 24;10(12):665. doi: 10.3390/v10120665 (PMC6315922; doi:10.3390/v10120665)
Supplement: Supplementary file 1 [file viruses-10-00665-s001.zip › SI/Table_S1.docx]

| **Isolate** | **Species** | **Year of isolation** | **Habitat** | **Location** | **Mycovirus** |
| --- | --- | --- | --- | --- | --- |
| EABa 10/01-Su | *Beauveria amorpha* | 2010 | Soil (sunflower field) | Córdoba (Spain) |  |
| EABb 00/11-Su | *Beauveria bassiana* | 2000 | Soil (scrubland) | Jaén (Spain) | + |
| EABb 00/13-Su | *Beauveria bassiana* | 2000 | Soil (woodland) | Jaén (Spain) | + |
| EABb 00/23-Su | *Beauveria bassiana* | 2000 | Soil (meadow) | Tenerife (Spain) |  |
| EABb 00/26-Su | *Beauveria bassiana* | 2000 | Soil (meadow) | Badajoz (Spain) |  |
| EABb 01/110-Su | *Beauveria bassiana* | 2001 | Soil (oak grove) | Sevilla (Spain) |  |
| EABb 01/112- Su | *Beauveria bassiana* | 2001 | Soil (wheat field) | Sevilla (Spain) | + |
| EABb 01/12-Su | *Beauveria bassiana* | 2001 | Soil (scrubland) | Sevilla (Spain) | + |
| EABb 01/171-Su | *Beauveria bassiana* | 2001 | Soil (cotton field) | Huelva (Spain) |  |
| EABb 01/33-Su | *Beauveria bassiana* | 2001 | Soil (olive grove) | Cadiz (Spain) | + |
| EABb 04/01-Tip | *Beauveria bassiana* | 2004 | *Iraella luteipes* (Himenoptera: Cinipidae) | Sevilla (Spain) |  |
| EABb 04/04-Su | *Beauveria bassiana* | 2004 | Soil (grassland) | Madrid (Spain) |  |
| EABb 07/06-Rf | *Beauveria bassiana* | 2007 | *Rhynchophorus ferrugineus* (Coleoptera: Curculionidae) | Alicante (Spain) | + |
| EABb 09/04-Su | *Beauveria bassiana* | 2009 | Soil (oak grove) | Ciudad Real (Spain) |  |
| EABb 09/06-Su | *Beauveria bassiana* | 2009 | Soil (eucalyptus grove) | Ciudad Real (Spain) |  |
| EABb 09/07-Fil | *Beauveria bassiana* | 2009 | Phylloplane (meadow) | Málaga (Spain) | + |
| EABb 09/11-Su | *Beauveria bassiana* | 2009 | Soil (olive grove) | Málaga (Spain) |  |
| EABb 09/16-Su | *Beauveria bassiana* | 2009 | Soil (olive grove) | Málaga (Spain) |  |
| EABb 09/31-Fil | *Beauveria bassiana* | 2009 | Phylloplane (oak grove) | Sevilla (Spain) |  |
| EABb 09/42-Fil | *Beauveria bassiana* | 2009 | Phylloplane (oak grove) | Sevilla (Spain) |  |
| EABb 10/01-Fil | *Beauveria bassiana* | 2010 | Phylloplane (olive grove) | Málaga (Spain) | + |
| EABb 10/103-Fil | *Beauveria bassiana* | 2010 | Phylloplane (olive grove) | Córdoba (Spain) |  |
| EABb 10/108-Su | *Beauveria bassiana* | 2010 | Soil (olive grove) | Córdoba (Spain) |  |
| EABb 10/111-Fil | *Beauveria bassiana* | 2010 | Phylloplane (meadow) | Málaga (Spain) |  |
| EABb 10/118-Fil | *Beauveria bassiana* | 2010 | Phylloplane (meadow) | Córdoba (Spain) |  |
| EABb 10/147-Fil | *Beauveria bassiana* | 2010 | Phylloplane (meadow) | Sevilla (Spain) |  |
| EABb 10/18-Su | *Beauveria bassiana* | 2010 | Soil (olive grove) | Córdoba (Spain) |  |
| EABb 10/27-Fil | *Beauveria bassiana* | 2010 | Phylloplane (meadow) | Málaga (Spain) |  |
| EABb 10/28-Su | *Beauveria bassiana* | 2010 | Soil (olive grove) | Córdoba (Spain) | + |
| EABb 10/30-Fil | *Beauveria bassiana* | 2010 | Phylloplane (olive grove) | Córdoba (Spain) | + |
| EABb 10/57-Fil | *Beauveria bassiana* | 2010 | Phylloplane (meadow) | Córdoba (Spain) | + |
| EABb 10/67-Fil | *Beauveria bassiana* | 2010 | Phylloplane (sunflower field) | Córdoba (Spain) |  |
| EABb 10/78-Fil | *Beauveria bassiana* | 2010 | Phylloplane (sunflower field) | Córdoba (Spain) |  |
| EABb 10/79-Fil | *Beauveria bassiana* | 2010 | Phylloplane (meadow) | Córdoba (Spain) |  |
| EABb 10/80-Fil | *Beauveria bassiana* | 2010 | Phylloplane (meadow) | Málaga (Spain) |  |
| EABpb 10/83-Fil | *Beauveria pseudobassiana* | 2010 | Phylloplane (meadow) | Málaga (Spain) |  |
| EABb 11/01-Mg | *Beauveria bassiana* | 2011 | *Monochamus galloprovincialis* (Coleoptera: Cerambycidae) | Palencia (Spain) | + |
| EABb 12/03-Pa | *Beauveria bassiana* | 2012 | *Paysandisia archon* (Lepidoptera: Castniidae) | Montpellier (France) |  |
| EABb 90/2-Dm | *Beauveria bassiana* | 1990 | *Dociostaurus maroccanus* (Orthoptera: Acrididae) | unknown |  |
| EABb 91/6-Ci | *Beauveria bassiana* | 1991 | *Calliptamus italicus* (Orthoptera: Acridide) | unknown |  |
| EABb 91/7-Dm | *Beauveria bassiana* | 1991 | *Dociostaurus maroccanus* (Orthoptera: Acrididae) | unknown |  |
| EABb 92/10-Dm | *Beauveria bassiana* | 1992 | *Dociostaurus maroccanus* (Orthoptera: Acrididae) | unknown |  |
| EABb 93/14-Tp | *Beauveria bassiana* | 1993 | *Thaumetopoea pytiocampa* (Lepidoptera: Notodontidae) | unknown |  |
| EABps 10/01-Su | *Beauveria pseudobassiana* | 2010 | Soil (oak grove) | Sevilla (Spain) |  |
| EABps 10/02-Su | *Beauveria pseudobassiana* | 2010 | Soil (oak grove) | Sevilla (Spain) |  |
| EABps 10/03-Su | *Beauveria pseudobassiana* | 2010 | Soil (oak grove) | Sevilla (Spain) |  |
| EABps 10/04-Su | *Beauveria pseudobassiana* | 2010 | Soil (oak grove) | Sevilla (Spain) |  |
| EABv 09/01-Su | *Beauveria varroae* | 2009 | Soil (oak grove) | Sevilla (Spain) |  |
| EABv 09/02-Su | *Beauveria varroae* | 2009 | Soil (oak grove) | Sevilla (Spain) |  |
| EABv 10/01-Su | *Beauveria varroae* | 2010 | Soil (oak grove) | Sevilla (Spain) |  |
| EAIf 10/01-Msp | *Isaria farinosa* | 2010 | *Monochamus sp.* | Palencia (Spain) |  |
| EALa 11/01-Msp | *Lecanicillium attenuatum* | 2011 | *Monochamus sp.* | Palencia (Spain) |  |
| EALa 12/01-Pa | *Lecanicillium attenuatum* | 2012 | *Paysandisia archon* | Catania (Italy) |  |
| EAMa 01/121-Su | *Metarhizium anisopliae* | 2001 | Soil (cotton field) | Sevilla (Spain) |  |
| EAMa 01/152-Su | *Metarhizium anisopliae* | 2001 | Soil (cotton field) | Sevilla (Spain) |  |
| EAMa 01/158-Su | *Metarhizium robertsii* | 2001 | Soil (olive grove) | Sevilla (Spain) |  |
| EAMa 01/58-Su | *Metarhizium brunneum* | 2001 | Soil (wheat field) | Córdoba (Spain) |  |
| EAMa 04/01-Ci | *Metarhizium anisopliae* | 2004 | *Calliptamus italicus* | Tbilisi (Georgia) |  |
| EAMa 06/01-Ct | *Metarhizium anisopliae* | 2006 | *Capnodis tenebrionis* | Apulia (Italy) |  |
| EAMa 08/01-Rf | *Metarhizium anisopliae* | 2008 | *Rhynchophorus ferrugineus* | Maglie (Italy) |  |
| EAMa 08/02-Rf | *Metarhizium anisopliae* | 2008 | *Rhynchophorus ferrugineus* | Brindisi (Italy) |  |
| EAMa 10/02-Fil | *Metarhizium anisopliae* | 2010 | Phylloplane (meadow) | Córdoba (Spain) |  |
| EAMa 10/05-Su | *Metarhizium guizhouense* | 2010 | Soil (olive grove) | Córdoba (Spain) |  |
| EAMa 10/06-Su | *Metarhizium guizhouense* | 2010 | Soil (olive grove) | Córdoba (Spain) |  |
| EAMb 09/01-Su | *Metarhizium brunneum* | 2009 | Soil (oak grove) | Sevilla (Spain) |  |
| EAMr 09/01-Su | *Metarhizium robertsii* | 2009 | Soil (oak grove) | Sevilla (Spain) |  |
| EAPl 10/01-Fil | *Purpureocillium lilacinum* | 2010 | Phylloplane (meadow) | Córdoba (Spain) |  |
| EAPl 10/01-Su | *Paecilomyces marquandii* | 2010 | Soil (olive grove) | Málaga (Spain) |  |
| EAPl 10/02-Fil | *Purpureocillium lilacinum* | 2010 | Phylloplane (meadow) | Córdoba (Spain) |  |
| EAPl 10/13-Su | *Purpureocillium lilacinum* | 2010 | Soil (olive grove) | Córdoba (Spain) |  |
| EAPl 10/14-Su | *Purpureocillium lilacinum* | 2010 | Soil (olive grove) | Córdoba (Spain) |  |
| EAPl 10/16-Su | *Purpureocillium lilacinum* | 2010 | Soil (olive grove) | Málaga (Spain) |  |
| EAPl 16/01-Su | *Purpureocillium lilacinum* | 2016 | Soil (sunflower field) | Córdoba (Spain) |  |
| EAPl 16/02-Su | *Purpureocillium lilacinum* | 2016 | Soil (sunflower field) | Cadiz (Spain) |  |
| EAPl 16/03-Su | *Purpureocillium lilacinum* | 2016 | Soil (sunflower field) | Sevilla (Spain) |  |
| KVL12-28 | *Metarhizium flavoviride* | 2009 | Soil; baited with Tenebrio molitor | Denmark |  |
| KVL12-29 | *Metarhizium majus* | 2009 | Soil; baited with Tenebrio molitor | Denmark |  |
| KVL12-30 | *Metarhizium brunneum* | 2009 | Soil; baited with Tenebrio molitor | Denmark |  |
| KVL12-32 | *Metarhizium robertsii* | 2009 | Soil; baited with Tenebrio molitor | Denmark |  |
| KVL12-35 | *Metarhizium robertsii* | 2009 | Soil; baited with Tenebrio molitor | Denmark |  |
| KVL12-36 | *Metarhizium robertsii* | 2009 | Soil; baited with Tenebrio molitor | Denmark |  |
| KVL12-37 | *Metarhizium brunneum* | 2009 | Soil; baited with Tenebrio molitor | Denmark |  |
| KVL03-122 | *Beauveria bassiana* | 2002 | Fly *Pegoplata aestiva* (Diptera: Anthomyiidae) | Denmark |  |
| KVL03-144 | *Beauveria bassiana* | 2002 | Grass bug *Leptopterna dolobrata* (Hemiptera: Miridae) | Denmark |  |
