# Supplementary material for: Mycoviral Population Dynamics in Spanish Isolates of the Entomopathogenic Fungus Beauveria bassiana
Source: Viruses. 2018 Nov 24;10(12):665. doi: 10.3390/v10120665 (PMC6315922; doi:10.3390/v10120665)
Supplement: Supplementary file 1 [file viruses-10-00665-s001.zip › SI/Table_S2.docx]

**Table S2.** Oligonucleotide primers used for viral sequence amplification.

| **Name** | **Sequence** | **T_m_** | **Amplicon** | **Ref.** |
| --- | --- | --- | --- | --- |
| BbVV1.F | 5’-AGACCCGTGCAATCTTCGC-3’ | 60^o^C | BbVV-1-like RdRp;  436 bp | [6] |
| BbVV1.R | 5’-TACACATCATCACCGGTGTG-3’ | 58^o^C |  | [6] |
| BbPV2.F | 5’-CGTGTCTGGCGGGTCAGCG-3’ | 66^o^C | BbPV-2-like RdRp;  787 bp | this study |
| BbPV2.R | 5’-CGCCACCTAACCACAGGCC-3’ | 64^o^C |  | this study |
| BbPmV1.F | 5’-CTGAGGGGCTAGATGCGATG-3’ | 63^o^C | BbPmV-1-like RdRp;  876 bp | this study |
| BbPmV1.R | 5’-CATGCCGACTTCCGATAGAAG-3’ | 61^o^C |  | this study |
